# Citric Acid Extraction Impact on Chemical and Bioavailable Forms of Metals in Soil

**DOI:** 10.3390/molecules30224480

**Published:** 2025-11-20

**Authors:** Krzysztof Barbusiński, Beata Karwowska, Ewa Neczaj

**Affiliations:** 1 Department of Water and Wastewater Engineering, Faculty of Energy and Environmental Engineering, Silesian University of Technology, Konarskiego St. 18A, 44-100 Gliwice, Poland; krzysztof.barbusinski@polsl.pl; 2 Department of Environmental Engineering and Biotechnology, Faculty of Infrastructure and Environment, Czestochowa University of Technology, Dąbrowskiego St. 69, 42-201 Częstochowa, Poland; ewa.neczaj@pcz.pl

**Keywords:** soil, heavy metals, BCR sequential extraction procedure, bioavailability, mobility, citric acid

## Abstract

The presence of heavy metals in soils poses a serious threat due to these harmful elements being transported into the food chain. The aim of the presented research was to evaluate the effect of the extraction of selected heavy metals from soil with a 1 M aqueous solution of citric acid (CA) on the chemical (including mobile) forms of these elements and their bioavailability. A soil sample taken in an industrial area was extracted. Then the total content of selected heavy metals (Pb, Zn, Cu, Cd, Ni), their chemical forms (determined by sequential extraction according to the BCR procedure) and bioavailable forms (determined by one-step extraction with a 1 M HCl) were determined in both types of soil, before and after extraction. The tested soil contained significant amounts of the tested metals, and the amounts can be compared as follows: Pb ≥ Zn > Cu ≥ Cd > Ni. The greatest threat to the environment is associated with the presence of cadmium and lead in the tested soil, the content of which exceeds the limits set for soils in industrial areas. In addition, the level of presence of heavy metals in bioavailable and mobile chemical forms, was considered significant. No clear correlation was observed between the content of the analyzed metals in mobile and bioavailable forms. The tested soil contained significant concentrations of Pb (2141 mg/kg), Zn (2030 mg/kg), Cu (68 mg/kg), Cd (63 mg/kg), and Ni (23 mg/kg), which were reduced to 857, 589, 42, 28, and 14 mg/kg, respectively, after extraction with 1 M CA. The extraction process with a CA solution reduced the content of all metals, and the efficiency of the process can be compared as follows: Zn > Pb > Cd > Ni ≥ Cu, with efficiencies of 71%, 60%, 55%, 41% and 39%, respectively. The extraction process reduced the metal content of all the bioavailable and chemical fractions. The shares of metals in the mobile fractions decreased in favor of the immobilized fractions and ones more stable in the environment. After the process of leaching metals from the soil, a clear tendency towards equalization of the heavy metal content in the mobile and bioavailable fractions was observed.

## 1. Introduction

The terrestrial environment, including soils, is an important part of the ecosystem that is the basis for the life processes of living organisms. Soil contaminants can be taken up during plant growth and easily penetrate the food chain [1]. Soil contamination with heavy metal compounds is a significant problem due to their potential harmfulness, persistence and tendency to accumulate in the tissues of living organisms. For these reasons, these substances are considered to cause significant health risk to the human population [2]. In addition, the presence of substances with a high tendency to accumulate can disrupt soil structure, reduce soil fertility and thus interfere with plant growth and development [3,4,5].

Sources of heavy metals in soils can be both natural and anthropogenic [1]. The natural processes through which metal compounds enter soils include volcanic eruptions, mineral weathering and erosion [1,6]. The sources resulting from human activity include, among others, industry (mining, metallurgy, and electroplating), sewage disposal, and agriculture (the use of fertilizers and pesticides) [7]. Heavy metal compounds accumulate in the surface layers of the soil, and can then migrate deep into the soil profile, potentially penetrating groundwater and traveling further in the environment. Additionally, due to weathering and leaching, they can be carried with soil particles to surface waters and enter the trophic chain [8]. Threats to living organisms are directly related to the amount of elements available to the biota and the amount reaching the bloodstream of people and animals [9].

Some heavy metals, such as Cu and Zn, are essential micronutrients in small amounts for metabolic processes, but in higher concentrations they can cause health problems, as well [10]. Metals such as Pb and Cd, on the other hand, are listed among the most toxic, having harmful effects on the human body even in trace amounts. They can cause disorders of important human body systems, such as cardiovascular, nervous, immune and reproductive [2,11], and have mutagenic and carcinogenic effects [4,12].

Sequential analysis provides important and valuable information on the mobility of metals, their availability to living organisms, and potential threats to the environment, which is much more detailed and informative than that derived from determining the total content of these elements [13,14]. The form of contaminants (chemical and bioavailable forms) is strongly related not only to the nature of the pollutants themselves, but also to environmental parameters, which include surface adsorption processes, pH and the presence of other substances [15,16]. The speciation and bioavailable forms of metals depend primarily on how these elements bind in the soil, exist in ionic form in the soil solution, and adsorb on the surface of mineral or organic matter. Additionally, they are associated with processes such as precipitation or complexation [17]. Sequential chemical extraction can be used to determine the proportion of chemical forms of metals in the total content of the element. Such an analysis involves conducting several consecutive extractions of metals from a solid matrix using solutions of chemical compounds in several stages with increasing strength to leach metals from the matrix [18]. The sequential analysis procedure results in a series of metal fractions with increasing strength to bind to the solid substrate. The fractions obtained from the first stage of the process are highly mobile, easily released from the substrate and can move freely in the ecosystem. The subsequent fractions are bound more and more strongly, and the last fraction of metals, usually called the residual fraction, includes metals permanently bound to the matrix. One of the first metal fractionation methods was proposed by Tessier et al. [19] for analyzing heavy metals in bottom sediments. The method has been modified multiple times, including by Sposito et al. [20] and Rudd et al. [21]. Currently, the one most widely used is BCR [22,23]. It allows to separation the total metal content into the following fractions: I—exchangeable and carbonate-bound metals (acid-soluble fraction), II—bound to iron and manganese oxides (reducible fraction), III—bound to organic matter and sulfides (oxidizable fraction), and IV—metals bound to silicates (residual fraction). The exchangeable and carbonate-bound forms are considered mobile and readily available for uptake. The metals bound to the iron and manganese oxide fractions, as well as organic and sulfide fractions, are considered temporarily immobilized and potentially immobile, while the residual ones are described as stable and immobile [23].

Bioavailability, as a parameter used in environmental risk assessment, should be measurable and quantifiable. However, there is no single consistent and practical methodology for determining bioavailability that provides unequivocal results for different organisms and situations encountered in the environment. There are currently two primary approaches to determining bioavailability. The first one involves the use of chemical methods of evaluation outside living organisms. In this case, the most commonly used processes involve the one-step extraction of metals from the test material using acid or salt solutions or metal complexing solutions [24,25]. The second type involves the use of biological tests based on the processes taking place inside the cells of living organisms [26].

Chemical extraction can be an effective method for reducing the total content of metals in soils, but it can also alter their speciation in the solid matrix and limit their mobility and bioavailability. Removal of metals from soil reduces the risks associated their potential penetration and transport in the environment. Leaching of metals from a solid sediment matrix is possible using a variety of processes, among which extraction using chelating agents or organic multi-carboxylic acids is very popular [27,28,29].

The purpose of this study was to evaluate the effect of extraction with a 1 M aqueous solution of citric acid (CA) on the total content of selected metals (Pb, Zn, Cu, Cd, and Ni) in soil taken from an industrial area, as well as on their chemical forms, including mobile and bioavailable ones, and to assess the potential environmental risk associated with their presence in different fractions. In this regard, citric acid was intentionally selected as a naturally occurring, biodegradable organic acid, which is consistent with the tenets of green chemistry and offers a more environmentally benign alternative to synthetic chelating agents commonly employed in soil remediation practices. By combining a detailed speciation approach (BCR sequential extraction) with bioavailability assessment, this study provides new insights into how such an environmentally friendly extraction method can not only reduce the overall content of heavy metals in soil but also shift their distribution from mobile to more stable forms. Chelating agents, such as EDTA, are more persistent in environment and their biodegradation is slower than citric acid (CA). This significantly limits the possibility of using EDTA for extracting of contaminants, such as heavy metals, from the soil. The use of CA is more advantageous and ensures faster removal of introduced chemicals from the soil. From the perspective of sustainable development, the findings highlight the potential of citric acid as an effective and environmentally friendly tool for mitigating the risks associated with heavy metals, while enabling the safe use of soil resources.

## 2. Materials and Methods

All chemicals used in this study were of analytical grade and obtained from recognized commercial suppliers. Citric acid monohydrate (≥99.5%) was purchased from Merck (Darmstadt, Germany). Hydrochloric acid (HCl, 37%) and nitric acid (HNO_3_, 65%) were obtained from Avantor Performance Materials (Gliwice, Poland). Reagents for the BCR sequential extraction procedure, including acetic acid (≥99.7%), hydroxylamine hydrochloride (≥99%), hydrogen peroxide (30%), and ammonium acetate (≥99%), were supplied by Sigma-Aldrich (St. Louis, MO, USA). All solutions were prepared using ultrapure water (resistivity 18.2 MΩ·cm) obtained from a Milli-Q purification system (Millipore, Bedford, MA, USA).

### 2.1. Materials and Methodology

The materials analyzed were soil samples collected in March 2025 near a zinc and lead smelter located near the town of Miasteczko Śląskie, Poland. Soil samples were collected from the 0–20 cm layer at five evenly distributed points within the industrial area. These materials can be classified as samples from an industrial area. The collected soil samples were mixed and air-dried under laboratory conditions (temperature of ca. 20 °C). The soil was then sifted through a sieve with a 0.4 mm mesh size of and homogenized into a composite sample to ensure representativeness. Until analysis, the soil samples were sealed tightly in plastic containers and stored in a refrigerator. The soil characteristics are presented in Table 1.

Determination of parameters of the analyzed soil samples was carried out using the following methods: pH in aqueous extract and in a 1 M KCl—by potentiometric method, while moisture content, total solids (TS), fixed solids (FS) and volatile solids (VS)—by weight method.

The content of bioavailable forms of metals was determined according to the procedure outlined by Snape et al. and Karczewska et al. [25,30] using a one-step extraction with a 1 M aqueous HCl solution carried out over 4 h under laboratory conditions.

The proportion of chemical forms of metals, including mobile ones, in soil samples was determined by sequential extraction analysis according to the BCR procedure [18,22,23].

The total metal content of the solid samples was determined after subjecting the soil material to microwave mineralization using a mixture of concentrated nitric acid (HNO_3_) and hydrochloric acid (HCl) at a volume ratio of 1:3, in an Easy ETHOS mineralizer by Milestone, Sorisole, Italy.

The metal content in liquid samples (after mineralization as well as after steps of BCR procedure) was determined using atomic absorption spectrometry (AAS) with a novAA 400 spectrometer by Analytik Jena, Jena, Germany.

The analyses were performed in three repetitions.

In the first stage of the study, part of the test material was extracted using a 1 M aqueous solution of citric acid (CA). Soil samples of 5 g were subjected to leaching with 50 mL of extractant solution over 6 h under laboratory conditions. After extraction, the samples were filtered through a paper filter and washed 3 times with distilled water to rinse away residual CA. The samples were then dried at ambient temperature (ca. 20 °C), crushed in a mortar and stored in plastic containers in the refrigerator until analysis. The use of a solution of a naturally occurring substance in the environment as an extractant greatly facilitates the post-process management of spent solutions, which, in accordance with the principles of sustainable development, can be reused or biodegraded after the possible recovery of metals.

The second stage of the research involved determining the characteristic parameters of the analyzed soil samples both before and after extraction with the CA solution. The determined parameters included pH, moisture content, TS, FS, VS and total content of selected heavy metals (lead, zinc, copper, cadmium and nickel) after microwave digestion.

In the third stage of the research, soils were analyzed both before and after CA extraction for bioavailable metals. For this purpose, 5 g soil samples were shaken with 50 mL of 1 M aqueous HCl solution at ambient temperature (about 20 °C) for four hours. The samples were then filtered through a paper filter into plastic flasks. The filtrate was analyzed for metal content.

**Figure 1:**
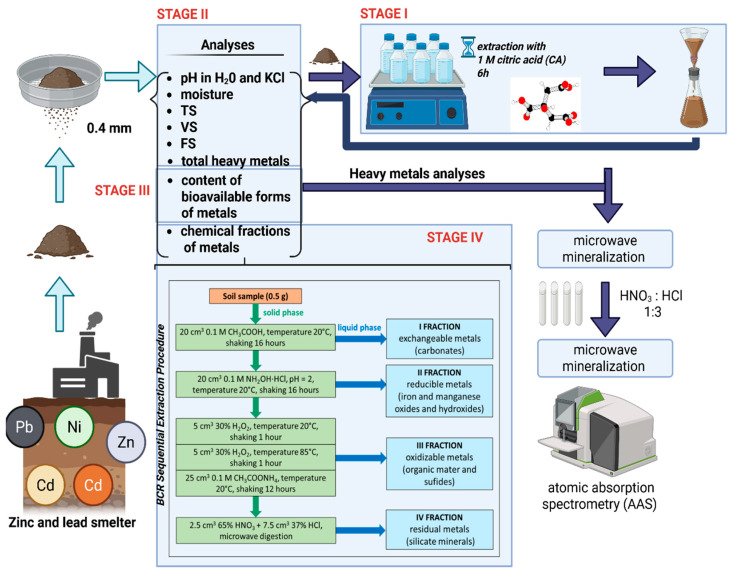
Scheme of experiment with details of BCR procedure for metal sequential analysis in two types of soil: before and after citric acid (CA) extraction.

In the fourth stage of the study, soils, both before and after CA extraction, were subjected to speciation analysis according to the BCR procedure [22,23] (Figure 1). The proportion of selected heavy metals in all four fractions of this procedure was determined, i.e., fraction I (FI)—exchangeable and carbonate-bound metals, fraction II (FII)—metals bound to hydrated iron and manganese oxides, fraction III (FIII)—metals bound to organic matter and sulfides, and fraction IV (FIV)—residual metals bound to silicates. The sum of the FI and FII fractions was determined as the content of mobile forms, i.e., those that are easily transportable in the environment. The sum of the FIII and FIV fractions was treated as the content of metals virtually immobilized in the matrix. The described research scheme is presented in Figure 1.

The potential environmental risk associated with the mobility of heavy metals was assessed using the Risk Assessment Code (RAC). This index, initially proposed by Perin et al. [31], is based on the proportion of a given metal present in the most labile fractions of the sequential extraction procedure. Specifically, RAC is calculated as the percentage of the sum of the exchangeable (FI) and carbonate-bound (FII) fractions relative to the total concentration of the metal (Equation (1)):

(1) $$RAC\;(\%)=\frac{F_{I}+F_{II}}{\sum F_{i}}\times 100$$

where:

*F_I_*—metal concentration in exchangeable fraction,
*F_II_*—metal concentration in reducible fraction,
Σ*F_i_*—the total concentration of the metal (sum of all BCR fractions).

According to the classification proposed by Perin et al. (1985) [31], RAC values are interpreted as follows: <1%: no risk; 1–10%: low risk; 11–30%: medium risk; 31–50%: high risk; >50%: very high risk.

The extraction conditions (1 M citric acid, six hours, solid-to-liquid ratio 1:10) were selected based on previous studies [27,32,33], which showed that these parameters provide efficient removal of heavy metals while preserving soil structure. This work aimed to evaluate the effect of citric acid under environmentally relevant, literature-supported conditions rather than to optimize process parameters.

### 2.2. Statistical Analyses

Statistical analyses were performed using Statistica 13 (StatSoft Inc., Tulsa, OK, USA). One-way ANOVA was applied to compare soil physicochemical parameters and total metal contents before and after citric acid extraction. Two-way ANOVA was used to assess the combined effects of extraction and fraction (FI–FIV) on metal distribution. It should be emphasized that the application of one-way and two-way ANOVA in this study was not intended to optimize the extraction conditions, as only one extraction setup (1 M citric acid, 6 hours, solid-to-liquid ratio 1:10) was tested. Instead, ANOVA was employed to statistically confirm whether the differences observed before and after extraction were significant and to assess how the extraction process and soil fractions (FI–FIV) affected the distribution of metals. This approach allowed us to quantitatively verify the effects of citric acid treatment on total, chemical, and bioavailable metal contents, ensuring that the reported changes were not random but statistically significant. Principal component analysis (PCA) was conducted to visualize the differences between groups and identify variables that contribute to data variance. All measurements were performed in triplicate, and the results are expressed as mean ± standard deviation (SD) in the tables. Figures illustrate relative proportions or trends for clarity. Statistical significance was considered at *p* < 0.05.

## 3. Results and Discussion

The physical and chemical characteristics of the soil remained essentially unchanged after citric acid extraction, as indicated in Table 1. The findings were substantiated by the outcomes of a one-way analysis of variance (independent variable: extraction utilizing citric acid), which revealed the absence of statistically significant differences (*p* > 0.05) among the soil parameters evaluated before and after the extraction process Both pH values (in H_2_O and KCl), moisture levels, TS, FS, VS showed no meaningful variation. This observation suggests that the citric acid intervention did not substantially change the core physicochemical properties of the soil matrix, with its impact primarily directed towards the redistribution and extraction of heavy metals.

The soil collected from the industrial area had a slightly acidic reaction (pH in the aqueous extract 5.4, in KCl solution 5.3), which decreased slightly after the leaching process with 1 M CA (pH 5.2 and 5.0 in the aqueous extract and KCl solution, respectively). Soil pH is considered one of the most important factors determining the concentration of metals in soil solution, their mobility and availability to plants. Heavy metals tend to be more strongly bound to the solid matrix in soils with higher pH and are therefore more difficult to release into the environment [34]. An increase in hydrogen ion concentration affects the intensity of heavy metal activation, increasing their mobility and the proportion of exchangeable forms. Therefore, in highly acidic soils, the mobility of metallic elements is much higher than in neutral and alkaline soils. In acidic soils, mobile forms of metals predominate. The measured pH values in the soil before and after extraction with a CA solution may tentatively indicate a higher content of the tested metals corresponding to the fractions commonly considered mobile (FI and FII). The moisture content of both types of test soil had the value of ca. 7% (7.4 and 6.9% for soil before and after CA extraction, respectively). On the other hand, the low organic matter content in the tested soil samples is indicated by the significant values of the ignition residue parameter of 954.4 and 953.7 g/kg for the soil before and after CA extraction, respectively. Since soil organic matter strongly contributes to metal retention through complexation and sorption [35,36,37], its low level may additionally enhance the mobility and potential bioavailability of heavy metals in the tested samples.

Table 2 presents the values of the total content of selected heavy metals in the soil before and after extraction with a 1 M aqueous CA solution. A one-way analysis of variance (ANOVA) performed on these data showed statistically significant differences in the concentrations of all tested metals between groups B (before extraction) and A (after extraction) with citric acid. For Pb, Zn, Cu, Cd, and Ni, the F values were 95,080.1 (*p* < 0.001), 255,482.2 (*p* < 0.001), 2868.5 (*p* < 0.001), 18,375.0 (*p* < 0.001), and 13,537.5 (*p* < 0.001), respectively. In all cases, metal concentrations decreased significantly after extraction, confirming the substantial impact of this process. The most pronounced decrease in concentrations was observed for zinc and lead, indicating their high mobility and susceptibility to complexation by organic acids.

The obtained total metal contents were compared with the regulatory limits established by the Polish Regulation of the Minister of the Environment (2016) [38] and with average background concentrations reported for uncontaminated soils [7]. The total concentrations of Pb (2141.7 mg/kg), Zn (2030.5 mg/kg), and Cd (63.4 mg/kg) in the analyzed soil exceeded the permissible thresholds for industrial areas (500, 1000, and 15 mg/kg, respectively) by approximately four-, two-, and fourfold. These exceedances confirm the industrial origin of the contamination and indicate a considerable environmental risk associated with the studied site.

The tested soils contained most lead and zinc ions. This is likely the result of the activity of a nearby zinc and lead smelter. The concentrations of the respective metals analyzed in the tested soil changed in the following order: Pb ≥ Zn > Cu ≥ Cd > Ni and were as follows: 2141.7, 2030.5, 68.2, 63.4, and 23.1 mg/kg of soil. These contents are significant even for the soils in industrialized areas, and in the case of zinc, cadmium, and lead, they exceeded the limits set by the Regulation of the Minister of the Environment on the Method of Conducting the Assessment of Pollution of the Earth’s Surface, dated 1 September 2016 [38]. The process of extracting metals from soil using a 1 M CA solution resulted in a significant reduction in the total content of the analyzed metals to 856.7, 589.0, 41.6, 28.4 and 13.6 mg/kg soil for Pb, Zn, Cu, Cd and Ni, respectively. Analysis of these variations indicates that the highest removal efficiencies were obtained for zinc (71%) and lead (60%), slightly lower for cadmium (55%), while the least leached metals were nickel (41%) and copper (39%). However, after the leaching process, the labeled total metal contents of cadmium and lead still exceeded the limits established for the land in industrial areas.

This order of extraction is in line with the literature, which indicates that Cu and Ni are generally more resistant to leaching due to their stronger association with the mineral fraction of soils. In contrast, Cd is often the easiest to mobilize. For example, Ke et al. [32] reported Cd removal of 89.1% from smelter-contaminated soils, while Pb and Cu were extracted much less efficiently (26.8% and 14.2%, respectively) using 0.1 M citric acid at pH 5. The study results show higher removal of Pb and Zn than those of Ke et al., which can be attributed to both the higher acid concentration applied (1 M vs. 0.1 M) and the different soil matrix investigated.

It is also worth noting that citric acid, although less efficient than synthetic chelating agents such as EDTA, is characterized by a transient mobilization effect. Meers et al. [39] demonstrated that the mobilization of labile metal fractions by citric acid was transient, with half-lives ranging from 1.5 to 5.7 days, whereas EDTA induced a much more persistent effect. This difference is environmentally relevant, since short-lived mobilization limits the risk of secondary contamination. In this respect, our findings support the view that citric acid, although less aggressive than EDTA, provides an effective and more sustainable option for metal removal. Recent reviews confirm that organic acids, including citric acid, offer a balance between efficiency and environmental safety, positioning them as promising tools in green chemistry for soil remediation (Zhang et al. [33]).

Literature data presented by many researchers shows that the total content of heavy metals in soils, bottom sediments and sewage sludge, does not reflect the real threat to ecosystems associated with the presence and toxic properties of these elements [18,34,40]. A more comprehensive picture of the risks may be obtained by incorporating additional data on their bioavailability and the potential for environmental transportation of heavy metal compounds, which can be achieved through a one-step extraction or a multi-step sequential analysis procedure [41]. Knowledge of the form of metallic elements in the soil, as well as how they bind to inorganic and organic components of the soil, allows a much better assessment of the possibility of their uptake by plants [34].

**Figure 2:**
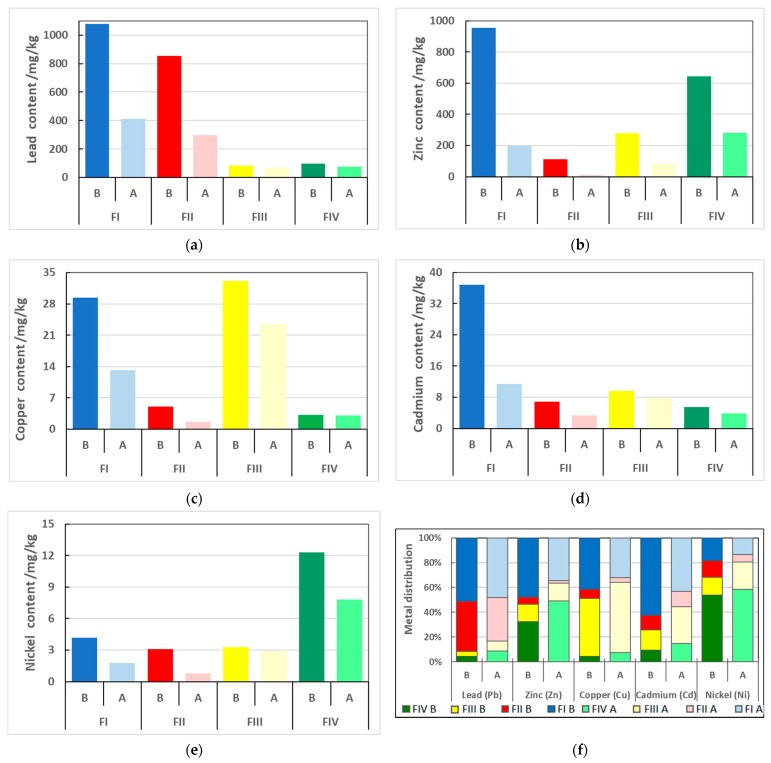
Changes in distribution of the analyzed heavy metals over the soil fractions before (B) and after (A) citric acid (CA) extraction. Fractions determined according to BCR sequential extraction procedure FI—exchangeable, FII—reducible, FIII—oxidizable, FIV—residual fraction: (**a**)—lead, (**b**)—zinc, (**c**)—copper, (**d**)—cadmium, (**e**)—nickel, (**f**)—percentage distribution changes.

Sequential analysis according to the BCR procedure showed that for soil not extracted with CA solution, a significant part of the metal content was part of the fractions considered mobile. The content of the respective examined metals in the soil fractions is summarized in Table 3, and their percentage distribution is shown in Figure 2.

The sum values represent calculated totals derived from averaged triplicate measurements; standard deviations were not propagated to prevent artificial overestimation of uncertainty. The overall uncertainty of total values was estimated below 5%, based on replicate analyses and instrumental precision.

The highest content of lead among the metals studied was recorded both in the soil taken directly from the environment and after the extraction process. As for most metals, in the case of lead, the proportion in the mobile FI fraction was the highest at 51.1% (1078.8 mg/kg of soil), slightly lower in the FII fraction at 40.5% (854.9 mg/kg of soil). The share of this metal in the FIII and FIV fractions was significantly lower and at similar levels. It amounted to, respectively: 3.9 and 4.5% (82.1 and 95.3 mg/kg of soil). Extraction with CA solution resulted in a slight decrease in the share of lead in the FI and FII fractions (48.2 and 35.0%) in favor of the fractions considered immobile FIII and FIV (7.9 and 8.8%). Literature data states that lead typically has a high affinity for the reducible (FII), oxidizable (FIII) and residual (FIV) fractions [18,40]. The significant proportion of the exchangeable fraction observed is most likely due to the environmental conditions in which the soil sample was collected.

In the case of zinc, 47.9% (954.2 mg/kg) of the total contents of all four fractions was located in the exchangeable fraction (FI), slightly less zinc—32.3% (642.7 mg/kg)—in the residual fraction (FIV) which is considered immobile in the environment. The other two soil fractions (FII—iron and manganese oxides fraction, and FIII—organic and sulfide fraction) contained far less of that metal, i.e., 5.6% (112.2 mg/kg) and 14.1% (280.1 mg/kg), respectively. The work of other researchers indicates that zinc typically binds to the fraction of iron and manganese oxides [18,42,43]. In the case of the soil collected in the field, the significant content of metal in the FI fraction is most likely the result of the acid reaction of the soil and the fact that zinc compounds are supplied to the soil as a result of the nearby smelter’s operation, and the stabilization processes in the soil matrix do not reach equilibrium.

The process of metal leaching with CA solution resulted in a reduction in zinc content in all fractions and in a change in the proportion of the respective chemical forms of this metal. After the metal extraction process, the proportion of zinc bound to mobile fractions (FI and FII) decreased in favor of the potentially immobilized fractions (FIII and FIV). The proportion of zinc in the FI and FII fractions decreased to 35% and 2%, respectively, while in the FIII fraction it remained steady at 14%, and in FIV it increased to 49%. Although much of the zinc was still contained in the fraction that moves most easily between elements of the environment, CA leaching resulted in much of the metal being contained in the fractions that do not pose a potential threat to the ecosystem.

Copper is a metal for which the most typical chemical form is the organic and sulfide fraction [18,40]. The soil tests conducted provided results consistent with those in the literature. Copper was located to the greatest extent in the FIII fraction, reaching a content of 46.8% (33.1 mg/kg of soil), while a slightly smaller share of 41.5% (29.4 mg/kg) was recorded in the exchangeable fraction (FI). The proportions of this metal in the other two fractions were significantly lower, at: 7.2% (5.1 mg/kg) and 4.5% (3.2 mg/kg) in FII and FIV, respectively. The leaching process with 1 M CA solution resulted in similar changes to those observed for the previously discussed metals. Proportions of copper increased in the FIII and FIV fractions and decreased in FI and FII. Ultimately, most copper was located in the organic and sulfide fraction (56.6%, 23.3 mg/kg of soil) and the exchangeable fraction (32.0%, 13.2 mg/kg of soil), slightly less in the residue fraction (7.5%, 3.1 mg/kg of soil), and least in the oxide fraction (3.9%, 1.6 mg/kg of soil).

Cadmium, the total content of which was similar to that of copper, mainly was bound to the most mobile exchangeable fraction (FI), in which its content was 36.8 mg/kg of the tested soil, accounting for 62.5% of its total content. Slightly less of that element was recorded in the FIII and FII fractions—9.7 mg/kg of soil (16.5%) and 6.9 mg/kg of soil (11.7%), respectively. The lowest percentage share was bound to the residual fraction (FIV), which accounted for 9.3% (5.5 mg/kg). As with zinc, the extraction process resulted in a decrease in the proportion of cadmium in the FI and FII fractions (43.0 and 12.5%) and an increase in the F III and FIV fractions (29.8 and 14.7%).

The total nickel content in the soil studied was the lowest of all the metals analyzed, and the distribution between the respective soil fractions was slightly different from that of the other metals. Its most significant proportion was recorded in the residue fraction, which considered virtually immobile in the environment. It amounted to 53.7% (12.3 mg/kg). A similar trend for nickel had been previously confirmed in the literature reports [18,40]. The remaining nickel was distributed more or less evenly among the other fractions: 18.4% (4.2 mg/kg), 14.4% (3.3 mg/kg) and 13.5% (3.1 mg/kg) for FI, FIII and FII, respectively. After the leaching process with the CA solution, the share of nickel in the residual fraction increased and still remained the largest: 58.7% (7.8 mg/kg of soil). The share in the organic and sulfide fraction also increased (21.8%, 2.9 mg/kg of soil). The shares of nickel in the FI and FII fractions dropped to 13.5% (1.8 mg/kg of soil) and 6.0% (0.8 mg/kg of soil), respectively.

The significant proportion of the metals in question in the most easily transported exchangeable fraction (FI) of the soil in the environment confirms the initial prediction based on the low pH value measured for the analyzed soil.

The summed content of metals in the four fractions determined by sequential extraction according to the BCR procedure represents 90 to 105% of the total metal content determined by complete microwave mineralization. Similar metal recovery results were achieved in earlier studies described in the literature [18,40].

Two-way ANOVA demonstrated statistically significant effects of both extraction (*p* < 0.05) and fraction (*p* < 0.01) on the distribution of all analyzed metals (Table 4). Moreover, the Extraction × Fraction interaction was significant (*p* < 0.05), indicating that the impact of citric acid varied between fractions. The most pronounced decreases were observed for Zn and Cu in FI and FII, while Pb and Cd remained associated mainly with these mobile fractions despite treatment. In contrast, Ni showed a partial shift toward the residual fraction (FIV). These findings highlight that citric acid selectively modifies the distribution of metals across geochemical pools, rather than uniformly reducing their concentrations a pattern consistent with earlier studies on sequential extraction and organic acid mobilization of heavy metals [44,45].

**Figure 3:**
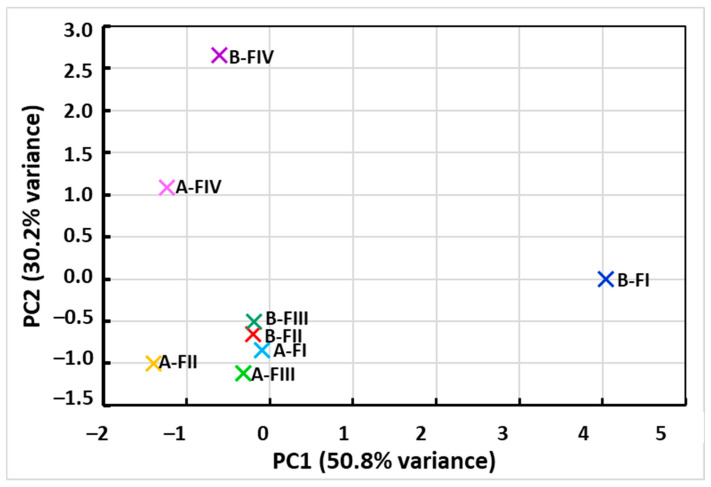
PCA loadings plot of heavy metals (PC1 is responsible for the extraction effect PC2 differentiates metals and fractions).

Principal component analysis (PCA) performed on average metal concentrations in individual fractions (FI–FIV) before and after citric acid extraction revealed a clear separation of samples between groups A and B in the space defined by the first two components. The first component (PC1), which explained the largest and most significant part of the variance, primarily reflected the effect of extraction and clearly separated the samples before and after the treatment. The second component (PC2) mainly differentiated individual metals and fractions, indicating their different binding properties in the tested matrix. The loadings plot (Figure 3) revealed that Pb and Zn had the most significant influence on the data structure, which is consistent with the ANOVA results, in which these metals were most susceptible to concentration reduction under the influence of citric acid. The PCA results therefore confirm that extraction not only significantly reduces the overall content of heavy metals, but also modifies their distribution between individual fractions, which is crucial for assessing the mobility and bioavailability of these elements.

**Figure 4:**
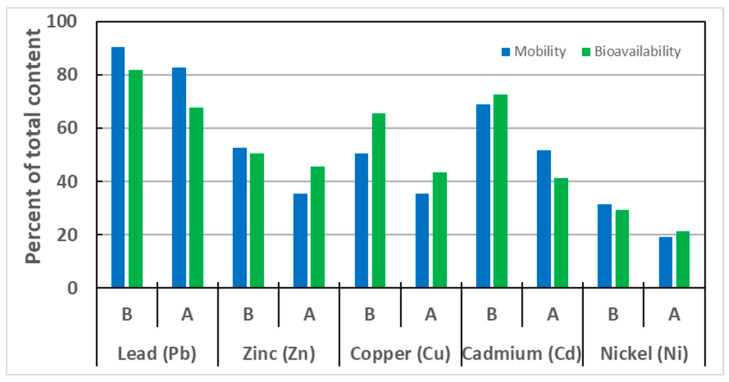
Percentage of the mobile and bioavailable form of metal in the total metal content in the soil fractions before (B) and after (A) CA extraction.

Metals associated with the FI and FII fractions are considered the most threatening forms to the environment, due to their high mobility and ability to be easily transported between different elements of the ecosystem [18,23]. Another method of assessing potential risks to living organisms, is the determination of bioavailable forms. Sometimes, both mobile and bioavailable forms are treated equivalently [13,46]. The content of mobile forms of metals in the studied soil, determined as the sum of the FI and FII fractions, as well as the content of the bioavailable forms of metals, determined by the single extraction method with 1 M HCl, are presented in Table 5, and their share in relation to the total content in Figure 4.

The characteristic feature of the studied soil was the location of most metals in the mobile fractions FI and FII, which are considered the fractions most susceptible to transport between environmental elements. The process of 1 M CA solution extraction resulted in a decrease in the proportion of mobile forms, with the largest decreases for Zn and Cd (by 17.1% and 17.2%, respectively), and slightly smaller ones for Cu and Ni (by 15.0% and 12.5%, respectively), and the smallest one for lead by 7%.

The shares of bioavailable forms of the analyzed metals at significant levels were also recorded ranging from about 30 to 80%. What is particularly concerning is the cases of lead and cadmium, where the percentage of bioavailable forms (respectively, 81.7 and 72.7%) coincides with the significant total content of these metals and mobile chemical forms recorded in the BCR analysis procedure. Zinc and copper had slightly lower bioavailability (50.6% and 65.5%, respectively). The lowest proportion of bioavailable forms was observed for nickel (29.4%), indicating that the stabilization processes of this metal in the soil matrix have been completed and reached a state of equilibrium. After extraction with the CA solution, the absolute contents of all metals in both mobile and bioavailable fractions decreased significantly. Their shares relative to the total content also reduced, but were not as pronounced as the contents expressed in mg/kg of soil.

The contents of the metals tested, recorded as the sum of the mobile fractions from sequential extraction and bioavailable fractions from a single HCl extraction, were not always consistent. The characteristic feature of lead, the absolute content of which was highest in the studied soil, was that the proportion of mobile and bioavailable forms, both before and after CA leaching, was significant. In this case, mobile forms prevailed over bioavailable ones. The CA leaching process had a much greater effect on reducing the content of bioavailable forms (by 14.1%) than mobile ones (by 7.4%).

For zinc, the content of mobile forms was slightly higher than that of bioavailable forms. After the extraction process with a CA solution, the content of bioavailable forms exceeded that of mobile forms. The extraction process significantly reduced the proportion of mobile forms (from 52.5 to 35.4%). The proportion of bioavailable forms was slightly less affected by extraction with a decrease in zinc content from 50.6% to 45.6% observed.

In contrast, the content of bioavailable copper fractions in soil before extraction is significantly higher than that of the fractions designated as mobile by sequential extraction. For soil subjected to leaching with the CA solution, the proportion of bioavailable forms is also higher than that of mobile forms. There is a significantly greater effect on reducing the content of bioavailable forms (by 22.0%) than on mobile forms (by 15.0%). After the extraction process, the content of both forms became almost equal, and it can be assumed that the amount of metal in the bioavailable form (18.1 mg/kg) coincides with the mobile forms (14.8 mg/kg).

In the case of cadmium, the bioavailable forms are virtually equivalent to the chemical forms bound to the first fraction and part of the second one. After extraction, it was observed that the proportion of bioavailable forms was significantly lower (41.2%) than the content of the metal’s mobile fraction (51.7%). The recorded reduction in the proportion of metal, as a result of the CA leaching process, was greater for the content of bioavailable than for mobile forms, and amounted to 31.5% and 17.2%, respectively.

For nickel, it was observed that the extraction process had little effect on the reciprocal arrangement of metal amounts in bioavailable and mobile forms. For both soil types, the shares of metals determined for the exchangeable and carbonate fractions were comparable to those determined to be bioavailable, with a slight predominance of mobile forms before extraction and bioavailable forms after the process. Quantitatively, the leaching process reduced the proportion of mobile forms by 12.5%, while that of bioavailable forms by 8.1%.

The relationship between the speciation and bioavailable forms of metals in contaminated soils has been shown previously in the literature [47]. However, the results generated from the presented work are not conclusive. Although the shares of mobile and bioavailable forms of metals in the soil studied were similar, for some metals (Zn, Cd, Cu) the content of bioavailable forms exceeds the content of mobile forms, and in other cases (Ni, Pb) the share of bioavailable forms is slightly lower. Therefore, the two forms should not be considered equal. Especially since, as reported by Wijayawardena et al. [9], the bioavailability of heavy metals depends on the type of soils, and a site-specific approach is necessary to determine this parameter accurately.

The two-way ANOVA revealed that both the type of fraction and the extraction step exerted a highly significant influence on Pb, Zn, and Cu concentrations, with strong interaction effects (Table 6). This indicates that the impact of citric acid extraction differed markedly between mobile and bioavailable fractions for these metals. For Cd, extraction was the dominant factor, while fraction had only a marginal effect (*p* ≈ 0.05). In contrast, Ni was affected exclusively by extraction, with neither fraction nor the interaction showing statistical significance. These outcomes suggest that Pb, Zn, and Cu exhibit a strong dependency on both soil fractionation and the extraction process, whereas Ni mobility is primarily determined by extraction itself.

**Figure 5:**
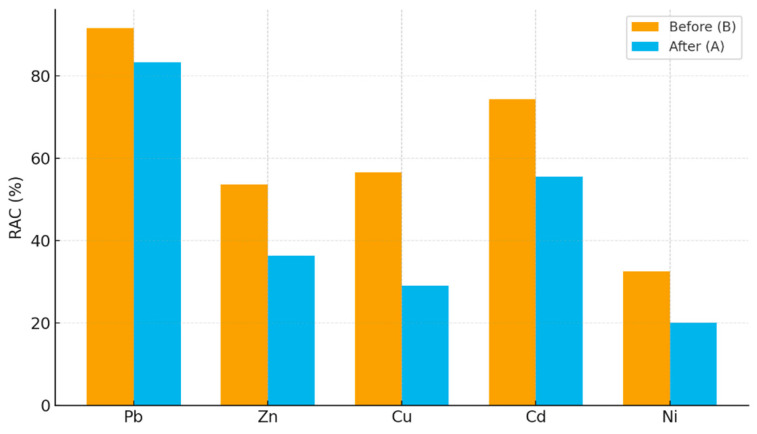
The risk assessment code: before v after citric acid extraction.

The RAC values (Figure 5) obtained in this study demonstrate that Pb and Cd remain in the high to very high risk category even after citric acid extraction, highlighting their strong association with labile fractions and persistent environmental risk. These findings align with earlier reports, which show that Pb and Cd often remain in exchangeable and carbonate fractions despite organic acid treatment, thereby sustaining long-term mobility [31,44]. In contrast, Zn and Cu exhibited pronounced reductions in RAC, shifting from very high to high or medium risk levels, respectively, which is consistent with previous studies demonstrating the enhanced mobilization of these metals by organic acids and their subsequent removal from bioavailable heavy metals pools [33,48]. Ni displayed intermediate behavior, with RAC values decreasing from high to medium risk, confirming observations by Pan et al. [49] that Ni tends to be less readily mobilized than Pb, Zn, or Cd.

Soil is an element of the environment that connects the links in the food chain. Contaminants found in soils, including heavy metals, can pose a potential threat to the natural environment because they can be easily transferred among organisms of the plant, animal and human worlds. This is particularly important when metals are in the forms that are easily transported in the environment and assimilated by living organisms. Thus, information on both the total content of these elements and their chemical forms, including mobile and bioavailable forms, is a very important aspect of soil characterization. A study and analysis of the distribution of the forms of the tested metals—lead, zinc, copper, cadmium, and nickel—in the soil samples taken from an industrial area revealed that the content of the analyzed metals in the tested soil was particularly high for lead and zinc. The extraction process using a 1 M aqueous CA solution resulted in a reduction in the total content of all metals, and those with the highest content by as much as 60–70%. The extraction of heavy metals from the soil and their transfer to a liquid medium provides a starting point for their recovery and use in industries where they do not pose a threat but are valuable raw materials, which is in line with the tenets of sustainable development.

Sequential analysis of the distribution of metal content in terms of chemical forms, carried out using the BCR method, showed that the greatest danger to the environment is associated with the presence of cadmium and lead in the soil, and to a slightly lesser extent, zinc. This soil was characterized by a low pH value and a preponderance of chemical forms of metals considered to be particularly easily transported in the environment, as well as a significant proportion of forms designated as bioavailable.

The process of extracting metals with a 1 M aqueous CA solution resulted in a decrease in the amount of metals in all fractions and a decrease in the proportion of metals in the mobile fractions (FI and FII) in favor of the potentially immobilized fractions (FIII and FIV). In addition, extraction resulted in equalization of the proportion of metals determined in mobile and bioavailable forms. The proportion of metal content in mobile and bioavailable forms was similar, but there is no clear correlation between these forms. Sometimes the content of bioavailable forms exceeds that of mobile forms. For this reason, it would be unjustified to translate the importance of mobile fractions of metals in the soil to their bioavailability. Determinations of mobile and bioavailable forms should be performed independently, using recognized and valid procedures.

Improving the quality of soils by removing harmful and toxic substances, including heavy metals, will enable safe and sustainable use of its resources both now and in the future.

## 4. Conclusions

This study successfully evaluated the impact of citric acid extraction on the chemical constituents and bioavailable forms of metals in soil. The results of the presented analyses demonstrated that citric acid, a biodegradable and naturally occurring organic acid, effectively reduced the total and mobile contents of heavy metals (Pb, Zn, Cu, Cd, Ni) in industrial soil. The extraction process not only decreased the total metal concentrations by up to 70% but also shifted their distribution from mobile and bioavailable fractions to more stable and environmentally safe forms.

Sequential analysis of the distribution of metal content in terms of chemical forms revealed that the greatest environmental risk was associated with the presence of cadmium and lead in the soil, and to a slightly lesser extent—zinc. This soil was characterized by a low pH value and a predominance of chemical forms of metals considered to be particularly easily transported in the environment, as well as a significant share of forms designated as bioavailable.

The process of extracting metals with a CA solution resulted in a decrease in the amount of metals in all fractions and a decrease in the proportion of metals in the mobile fractions (FI and FII) in favor of the potentially immobilized fractions (FIII and FIV). In addition, extraction resulted in equalization of the proportion of metals determined in mobile and bioavailable forms. The proportion of metal content in mobile and bioavailable forms was similar, but there is no clear correlation between these forms. Sometimes the content of bioavailable forms exceeds that of mobile forms. Therefore, it would be unjustified to translate the importance of mobile fractions of metals in the soil to their bioavailability. In the context of these results, it can be concluded that the determinations of mobile and bioavailable forms of metals should be performed independently, using recognized and validated procedures.

These findings highlight the environmental significance of citric acid extraction as a green and sustainable remediation method. By lowering the bioavailability and mobility of toxic metals, this process minimizes potential risks of soil-to-plant transfer and groundwater contamination. Moreover, the extracted metals can be recovered from the leachate, contributing to the principles of a circular economy and reducing waste generation.

The effectiveness of citric acid across varying soil conditions—particularly acidic and metal-rich industrial soils—indicates its potential for application in sustainable soil management and remediation strategies. This approach enables the safe reuse of treated soils in less sensitive areas, supporting the recovery of valuable metals and aligning with the principles of green chemistry and environmental protection.

## Figures and Tables

**Table 1:** Physicochemical characteristics of the soil before (B) and after (A) citric acid (CA) extraction.

Parameter		Soil	
		B	A
pH	H_2_O	5.4 ± 0.1	5.2 ± 0.1
	KCl	5.3 ± 0.2	5.0 ± 0.1
moisture, %		7.4 ± 0.4	6.9 ± 0.3
TS, g/kg		926 ± 1	931 ± 3
VS, g/kg		45.6 ± 0.3	46.3 ± 0.2
FS, g/kg		954.4 ± 0.2	953.7 ± 0.2

**Table 2:** Total heavy metal content in the soil before (B) and after (A) citric acid (CA) extraction.

Metal	Content, mg/kg Soil	
	B	A
Lead (Pb)	2141.7 ± 5.3	856.7 ± 4.9
Zinc (Zn)	2030.5 ± 4.2	589.0 ± 2.6
Copper (Cu)	68.2 ± 0.5	41.6 ± 0.7
Cadmium (Cd)	63.4 ± 0.2	28.4 ± 0.4
Nickel (Ni)	23.1 ± 0.1	13.6 ± 0.1

**Table 3:** The heavy metal content (mg/kg soil) in the fractions (FI–FIV) of the soil before (B) and after (A) citric acid (CA) extraction.

Fraction	Lead (Pb)		Zinc (Zn)		Copper (Cu)		Cadmium (Cd)		Nickel (Ni)	
	B	A	B	A	B	A	B	A	B	A
FI	1078.8 ± 2.2	411.2 ± 2.3	954.2 ± 4.4	198.6 ± 0.8	29.4 ± 0.2	13.2 ± 0.1	36.8 ± 0.4	11.4 ± 0.2	4.2 ± 0.2	1.8 ± 0.1
FII	854.9 ± 1.8	298.9 ± 1.1	112.2 ± 0.9	10.1 ± 0.3	5.1 ± 0.1	1.6 ± 0.1	6.9 ± 0.3	3.3 ± 0.1	3.1 ± 0.2	0.8 ± 0.1
FIII	82.1 ± 0.8	67.5 ± 0.3	280.1 ± 1.3	82.8 ± 0.7	33.1 ± 0.3	23.3 ± 0.3	9.7 ± 0.2	7.9 ± 0.2	3.3 ± 0.1	2.9 ± 0.1
FIV	95.3 ± 1.1	75.2 ± 0.5	642.7 ± 3.2	281.8 ± 2.0	3.2 ± 0.1	3.1 ± 0.2	5.5 ± 0.2	3.9 ± 0.1	12.3 ± 0.3	7.8 ± 0.2
Sum: FI–FIV	2111.1	852.8	1989.2	573.3	70.8	41.2	58.9	26.5	22.9	13.2

**Table 4:** Results of two-way ANOVA for the effect of Extraction (before [B], after [A]) and Fractionation (FI-FIV) on metal distribution.

Metal	Effect	df	F	*p*-Value	Significance
Pb	Extraction (A vs. B)	1	283,241.3	<0.001	***
	Fraction (FI–FIV)	3	335,510.8	<0.001	***
	Interaction	3	85,775.4	<0.001	***
Zn	Extraction (A vs. B)	1	161,155.4	<0.001	***
	Fraction (FI–FIV)	3	73,815.7	<0.001	***
	Interaction	3	26,724.4	<0.001	***
Cu	Extraction (A vs. B)	1	1000.0	<0.001	***
	Fraction (FI–FIV)	3	25,924.0	<0.001	***
	Interaction	3	4609.9	<0.001	***
Cd	Extraction (A vs. B)	1	7323.9	<0.001	***
	Fraction (FI–FIV)	3	9322.0	<0.001	***
	Interaction	3	3734.7	<0.001	***
Ni	Extraction (A vs. B)	1	1105.9	<0.001	***
	Fraction (FI–FIV)	3	2656.8	<0.001	***
	Interaction	3	134.7	<0.001	***

Legend: *p* < 0.001 (***).

**Table 5:** The heavy metal content (mg/kg soil) in the mobile (FI + FII) and bioavailable forms of the soil before (B) and after (A) citric acid (CA) extraction.

Form	Lead (Pb)		Zinc (Zn)		Copper (Cu)		Cadmium (Cd)		Nickel (Ni)	
	B	A	B	A	B	A	B	A	B	A
Mobile	1933.7 ± 4.4	710.1 ± 3.1	1066.4 ± 2.2	208.7 ± 0.8	34.5 ± 0.2	14.8 ± 0.2	43.7 ± 0.3	14.7 ± 0.1	7.3 ± 0.5	2.6 ± 0.3
Bioavailable	1749.3 ± 3.9	579.5 ± 2.5	854.9 ± 2.5	298.9 ± 1.1	65.5 ± 0.4	18.1 ± 0.2	46.1 ± 0.3	11.7 ± 0.1	6.8 ± 0.3	2.9 ± 0.5

**Table 6:** Results of two-way ANOVA for the effects of Fraction (Mobile, Bioavailable), Extraction (before [B], after [A]), and their interaction on metal concentration.

Metal	Factor	F	*p*-Value	Significance
Pb	Fraction	5902.7	<0.0001	***
	Extraction	340,771.2	<0.0001	***
	Fraction × Extraction	172.2	<0.0001	***
Zn	Fraction	3411.2	<0.0001	***
	Extraction	463,341.8	<0.0001	***
	Fraction × Extraction	21,102.7	<0.0001	***
Cu	Fraction	12,605.2	<0.0001	***
	Extraction	48,240.1	<0.0001	***
	Fraction × Extraction	8221.0	<0.0001	***
Cd	Fraction	5.4	0.049	*
	Extraction	60,293.4	<0.0001	***
	Fraction × Extraction	437.4	<0.0001	***
Ni	Fraction	2.2	0.176	n.s.
	Extraction	1754.2	<0.0001	***
	Fraction × Extraction	2.2	0.176	n.s.

Legend: *p* < 0.05 (*), *p* < 0.001 (***), n.s.—not significant.

## Data Availability

Data is contained within article.

## Abbreviations

The following abbreviations are used in this manuscript:

CA	Citric acid
TS	Total solids
FS	Fixed solids
VS	Volatile solids
AAS	Atomic absorption spectrometry
RAC	Risk Assessment Code
PCA	Principal component analysis

## References

[1] Chen H., Gao Y., Dong H., Sarkar B., Song H., Li J., Bolan N., Quin B.F., Yang X., Li F. (2023). Chitin and crawfish shell biochar composite decreased heavy metal bioavailability and shifted rhizosphere bacterial community in an arsenic/lead co-contaminated soil. Environ. Int..

[2] Giri S., Singh A.K. (2016). Spatial distribution of metal(loid)s in groundwater of a mining dominated area: Recognising metal(loid) sources and assessing carcinogenic and non-carcinogenic human health risk. Int. J. Environ. Anal. Chem..

[3] Chen H., Feng Y., Yang X., Yang B., Biony S., Bolan N.S., Meng J., Wong J.W.C., Wu F., Chen W. (2022). Assessing simultaneous immobilization of lead and improvement of phosphorus availability through application of phosphorus-rich biochar in a contaminated soil: A pot experiment. Chemosphere.

[4] Gul I., Manzoor M., Kallerhoff J., Arshad M. (2020). Enhanced phytoremediation of lead by soil applied organic and inorganic amendments: Pb phytoavailability, accumulation and metal recovery. Chemosphere.

[5] Jaskulak M., Grobelak A., Vandenbulcke F. (2020). Effects of sewage sludge supplementation on heavy metal accumulation and the expression of ABC transporters in *Sinapis alba* L. during assisted phytoremediation of contaminated sites. Ecotoxicol. Environ. Saf..

[6] Toth G., Hermann T., Szatmari G., Pasztor L. (2016). Maps of heavy metals in the soils of the European Union and proposed priority areas for detailed assessment. Sci. Total Environ..

[7] Rinklebe J., Shaheen S.M. (2014). Assessing the mobilization of cadmium, lead, and nickel using a seven-step sequential extraction technique in contaminated floodplain soil profiles along the Central Elbe River, Germany. Water Air Soil Pollut..

[8] Nowack B., Schulin R., Luster J. (2010). Metal fractionation in a contaminated soil after reforestation: Temporal changes versus spatial variability. Environ. Pollut..

[9] Wijayawardena M.A.A., Yan K., Liu Y., Naidu R. (2023). Can the mouse model successfully predict mixed metal(loid)s bioavailability in humans from contaminated soils?. Chemosphere.

[10] Setia R., Dhaliwal S.S., Singh R., Kumar V., Taneja S., Kukal S.S., Pateriya B. (2020). Impact assessment of metal contamination in surface water of Sutlej River (India) on human health risks. Environ. Pollut..

[11] Zuo T.-T., Li Y.-L., Wang Y., Guo Y.-S., Shen M.-R., Yu J.-D., Li J., Jin H.-Y., Wei F., Ma S.-C. (2023). Distribution, speciation, bioavailability, risk assessment, and limit standards of heavy metals in Chinese herbal medicines. Pharmacol. Res.–Mod. Chin. Med..

[12] Ahmad H.R., Mehmood K., Sardar M.F., Maqsood M.A., Rehman M.Z.U., Zhu C., Li H. (2019). Integrated risk assessment of potentially toxic elements and particle pollution in urban road dust of megacity of Pakistan. Hum. Ecol. Risk Assess..

[13] Bastami K.D., Neyestani M.R., Molamohyedin N., Shafeian E., Haghparast S., Shirzadi I.A., Baniamam M. (2018). Bioavailability, mobility, and origination of metals in sediments from Anzali Wetland, Caspian Sea. Mar. Pollut. Bull..

[14] Bastami K.D., Neyestani M.R., Esmaeilzadeh M., Haghparast S., Alavi C., Fathi S., Nourbakhsh S., Shirzadi E.A., Parhizgar R. (2017). Geochemical speciation, bioavailability and source identification of selected metals in surface sediments of the Southern Caspian Sea. Mar. Pollut. Bull..

[15] Nkoh J.N., Ajibade F.O., Atakpa E.O., Baquy M.A.-A., Mia S., Odii E.C., Xu R. (2022). Reduction of heavy metal uptake from polluted soils and associated health risks through biochar amendment: A critical synthesis. J. Hazard. Mater. Adv..

[16] Kumar V., Rout C., Singh J., Saharan Y., Goyat R., Umar A., Akbar S., Baskoutas S. (2023). A review on the clean-up technologies for heavy metal ions contaminated soil samples. Heliyon.

[17] Li Q., Wang Y., Li Y., Li L., Tang M., Hu W., Chen L., Ai S. (2022). Speciation of heavy metals in soils and their immobilization at micro-scale interfaces among diverse soil components. Sci. Total Environ..

[18] Dąbrowska L. (2012). Speciation of heavy metals in sewage sludge after mesophilic and thermophilic anaerobic digestion. Chem. Pap..

[19] Tessier A., Campbell P.G.C., Bisson M. (1979). Sequential extraction procedure for the speciation of particulate trace metals. Anal. Chem..

[20] Sposito G., Lund L.J., Chang A.C. (1982). Trace metal chemistry in arid-zone field soils amended with sewage sludge: I. Fractionation of Ni, Cu, Zn, Cd, and Pb in solid phases. Soil Sci. Soc. Am. J..

[21] Rudd T., Lake D.L., Mehrotra I., Sterritt R.M., Kirk P.W.W., Campbell J.A., Lester J.N. (1988). Characterization of metal forms in sewage sludge by chemical extraction and progressive acidification. Sci. Total Environ..

[22] Ure A.M., Quevauviller P.H., Muntau H., Griepink B. (1993). Speciation of heavy metals in soils and sediments. An account of the improvement and harmonization of extraction techniques undertaken under the auspices of the BCR of the Commission of the European Communities. Int. J. Environ. Anal. Chem..

[23] Rauret G., Lopez-Sanchez J.F., Sahuquillo A., Barahona E., Lachica M., Ure A.M., Davidson C.M., Gomez A. (2000). Application of a modified BCR sequential extraction (three-step) procedure for the determination of extractable trace metal contents in a sewage sludge amended soil reference material (CRM 483), complemented by a three-year stability study of acetic acid and EDTA extractable metal content. J. Env. Monit..

[24] Li Y., Rothwell S., Cheng H., Jones K.C., Zhang H. (2019). Bioavailability and metabolism in a soil-crop system compared using DGT and conventional extraction techniques. Environ. Int..

[25] Snape I., Scouller R.C., Stark S.C., Stark J., Riddle M.J., Gore D.B. (2004). Characterisation of the dilute HCl extraction method for the identification of metal contamination in Antarctic marine sediments. Chemosphere.

[26] Kumpiene J., Giagnoni L., Marschner B., Denys S., Mench M., Adriaensen K., Vangronsveld J., Puschenreiter M., Renella G. (2017). Assessment of methods for determining bioavailability of trace elements in soils: A review. Pedosphere.

[27] Di Palma L., Mecozzi R. (2007). Heavy metals mobilization from harbor sediments using EDTA and citric acid as chelating agents. J. Hazard. Mater..

[28] Zorpas A.A., Loizidou M. (2009). The application of inorganic and organic acids for the treatment of heavy polluted sewage sludge and the evaluation of the remaining metal with sequential extraction. Desalin. Water Treat..

[29] Nair A., Juwarkar A.A., Devotta S. (2008). Study of speciation of metals in an industrial sludge and evaluation of metal chelators for their removal. J. Hazard. Mater..

[30] Karczewska A., Kabała C. (2019). Metodyka Analiz Laboratoryjnych Gleb i Roślin (Methodology of Laboratory Analyzes of Soils and Plants).

[31] Perin G., Craboledda L., Lucchese M., Cirillo R., Dotta L., Zanetta M.L., Oro A., Lakkas T. (1985). Heavy metal speciation in the sediments of Northern Adriatic Sea: A new approach for environmental toxicity determination. Heavy Metals in the Environment.

[32] Ke X., Zhang F.J., Zhou Y., Zhang H.J., Guo G.L., Tian Y. (2020). Removal of Cd, Pb, Zn, Cu in smelter soil by citric acid leaching. Chemosphere.

[33] Zhang H., Xu Y., Kanyerere T., Wang Y.-s., Sun M. (2022). Washing reagents for remediating heavy-metal-contaminated soil: A review. Front. Earth Sci..

[34] Fijałkowski K., Kacprzak M., Grobelak A., Placek A. (2012). The influence of selected soils parameters on the mobility of heavy metals in soils. Inżynieria i Ochrona Środowiska.

[35] Yu H., Li C., Yan J., Ma Y., Zhou X., Yu W., Kan H., Meng Q., Xie R., Dong P. (2023). A review on adsorption characteristics and influencing mechanism of heavy metals in farmland soil. RSC Adv..

[36] Qi C., Hu T., Zheng Y., Wu M., Tang F.H.M., Liu M., Zhang B., Derrible S., Chen Q., Hu G. (2025). Global and regional patterns of soil metal(loid) mobility and associated risks. Nat. Commun..

[37] Angon P.B., Islam S., Kc S., Das A., Anjum N., Poudel A., Suchi S.A. (2024). Sources, effects and present perspectives of heavy metals contamination: Soil, plants and human food chain. Heliyon.

[38] Regulation of the Minister of the Environment of 1 September 2016 on the Method of Conducting the Assessment of Pollution of the Earth’s Surface, Dz.U.2016 poz. 1395. https://isap.sejm.gov.pl/isap.nsf/DocDetails.xsp?id=wdu20160001395.

[39] Meers E., Lesage E., Lamsal S., Hopgood M., Vervaeke P., Tack F.M.G., Verloo M.G. (2005). Enhanced phytoextraction: I. Effect of EDTA and citric acid on heavy metal mobility in a calcareous soil. Int. J. Phytoremed..

[40] Fuentes A., Lloréns M., Sáez J., Soler A., Aguilar M.I., Orutño J.F., Meseguer V.F. (2004). Simple and sequential extractions of heavy metals from different sewage sludges. Chemosphere.

[41] Bhattacharyya P., Tripathy S., Chakrabarti K., Chakraborty A., Banik P. (2008). Fractionation and bioavailability of metals and their impacts on microbial properties in sewage irrigated soil. Chemosphere.

[42] Chen M., Li X., Yang Q., Zeng G., Zhang Y., Liao D., Liu J., Hu J., Guo L. (2008). Total concentration and speciation of heavy metals in municipal sludge from Changsha, Zhuzhou and Xiangtan in middle—south region of China. J. Hazard. Mater..

[43] Jamali M.K., Kazi T.G., Arain M.B., Afridi H., Jalbani N., Kandhro G.A., Shah A.Q., Baig J.A. (2009). Speciation of heavy metals in untreated sewage sludge by using microwave assisted sequential extraction procedure. J. Hazard. Mater..

[44] Liang G., Zhang B., Lin M., Wu S., Hou H., Zhang J., Qian G., Huang X., Zhou J. (2017). Evaluation of heavy metal mobilization in creek sediment: Influence of RAC values and ambient environmental factors. Sci. Total Environ..

[45] Zhang S., Wang T., Wang H., Kang Q., Zhou Q., Chen B. (2022). Spatial pattern, sources identification, and risk assessment of heavy metals in a typical soda soil from Bayannur, Northwestern China. Int. J. Environ. Res. Public Health.

[46] Upama D., Bhattacharyya K.G. (2018). Mobility and bioavailability of Cd, Co, Cr, Cu, Mn and Zn in surface runoff sediments in the urban catchment area of Guwahati, India. Apll. Water Sci..

[47] Finzgar N., Tlustos P., Lestan D. (2007). Relationship of soil properties to fractionation, bioavailability and mobility of lead and zinc in soil. Plant. Soil Environ..

[48] Kumar V., Sahu P., Markandeya (2022). Sequential extraction and risk assessment of pollutants from one major tributary of the Ganga. Water Supply.

[49] Pan Y., Chen M., Wang X., Chen Y., Dong K. (2023). Ecological risk assessment and source analysis of heavy metals in the soils of a lead-zinc mining watershed area. Water.

